# The BCLF's core objectives, mission, and logo symbolism

**DOI:** 10.5937/jomb0-56486

**Published:** 2025-06-13

**Authors:** Nuray N. Ulusu, Nada Majkić-Singh

**Affiliations:** 1 Koc University, School of Medicine, Department of Medical Biochemistry, Istanbul, Turkey; 2 Koç University, Research Center for Translational Medicine (KUTTAM), Istanbul, Turkey; 3 Society of Medical Biochemists of Serbia, Belgrade

**Keywords:** Balkan clinical laboratory federation (BCLF), clinical biochemistry, logo design, regional collaboration, professional development, healthcare collaboration, educational events, Balkanska federacija kliničkih laboratorija (BCLF), klinička biohemija, dizajn logotipa, regionalna saradnja, profesionalni razvoj, saradnja u zdravstvu, edukativni događaji

## Abstract

The Balkan Clinical Laboratory Federation (BCLF) is important in advancing clinical laboratory practices and fostering collaboration across the Balkan region. This federation was established in 1992, the BCLF aims to unite medical biochemists from Balkan countries, promoting standardization, innovation, and professional development in clinical laboratory medicine. This personal view article explores the federation's growth, its impact on clinical biochemistry, and the process behind the creation of its logo. The BCLF's logo designed during a Congress in 2010 and accepted in 2011, symbolizes the unity and interconnectedness of the region. The blue section represents the sea, the orange symbolizes the Balkans, and the green section represents Turkey, collectively illustrating the federation's collaborative nature. The colors were chosen for their symbolic meanings: orange embodies optimism and enthusiasm, green signifies growth and new beginnings, and blue reflects grandeur and depth. The logo plays a significant role in visually representing the values and mission of the BCLF. The BCLF has successfully organized educational events, conferences, and workshops, facilitated knowledge exchange, and helped laboratory professionals stay current with new methodologies. Additionally, the federation advocates for improved healthcare policies and laboratory infrastructure, ensuring the sustainability of clinical laboratory services in the region. Through its dedication to unity, research, and knowledge sharing, the BCLF has elevated clinical biochemistry in the region, creating a foundation for continued growth and collaboration in the clinical biochemistry field.

## Abstract (sr)

Balkanska federacija kliničkih laboratorija (BCLF) ima ključnu ulogu u unapređenju praksi kliničkih laboratorija i podsticanju saradnje u okviru Balkanskog regiona. Ova federacija je osnovana 1992. godine, a njen cilj je da ujedini medicinske biohemičare iz balkanskih zemalja, promovišući standardizaciju, inovacije i profesionalni razvoj u oblasti kliničke laboratorijske medicine. Ovaj članak, zasnovan na ličnom stavu, istražuje rast federacije, njen uticaj na kliničku biohemiju i proces nastanka njenog logotipa. Logotip BCLF-a dizajniran je na kongresu 2010. godine i prihvaćen 2011. godine, simbolizujući jedinstvo i međusobnu povezanost regiona. Plavi deo predstavlja more, narandžasti simbolizuje Balkan, a zeleni deo predstavlja Tursku, što kolektivno ilustruje saradnički karakter federacije. Boje su odabrane zbog njihovih simboličkih značenja: narandžasta označava optimizam i entuzijazam, zelena simbolizuje rast i nove početke, dok plava odražava veličinu i dubinu. Logotip ima značajnu ulogu u vizuelnom predstavljanju vrednosti i misije BCLF-a. BCLF je uspešno organizovao edukativne događaje, konferencije i radionice, olakšavajući razmenu znanja i pomažući profesionalcima u laboratorijama da ostanu u koraku sa novim metodologijama. Pored toga, federacija se zalaže za unapređenje politika u zdravstvu i infrastrukture laboratorija, osiguravajući održivost kliničkih laboratorijskih usluga u regionu. Kroz posvećenost jedinstvu, istraživanju i deljenju znanja, BCLF je unapredio praksu kliničke biohemije u regionu, stvarajući temelj za nastavak rasta i saradnje u ovoj oblasti.

## Introduction

Nowadays logo detection is one of the crucial branches of computers which is different from the traditional handcraft methods [1]. The meaning of logos and the story behind them may be more interesting than expected. Logos are deeply rooted in human cultures, and history and carry various meanings. Each symbol offers rich historical events, ideas, relationships, cultures, society, objects, geographic areas, social dynamics, or belief systems [2].

In this context, exploring the meaning behind symbols enhances our understanding of the human experience and explains cultural interactions across cultures. However, logos may have meanings or stories everyone can see and understand [3]. But sometimes the logos have different stories. The Balkan Clinical Laboratory Federation (BCLF) is one of the most important federations in the Balkan region. This Federation began with the Yugoslav Society of Medical Biochemists on May 15, 1955, as a section within the Union of Pharmaceutical Societies of Yugoslavia. It became an independent organization on April 6, 1989, with its headquarters in Belgrade. The Society’s main goal is to unite medical biochemists across Yugoslavia and promote the development of medical biochemistry within the public healthcare system [4].

The BCLF has been a significant and longstanding federation, and the creation of its logo is a relatively recent development. This article explores the growth of the BBCLF and provides an in-depth look into the process of designing its logo. The logo, as a symbol, plays an essential role in representing the values and mission of the Federation. It also highlights the importance of unity among the Balkan countries, reflecting their shared goals and commitment to advancing clinical biochemistry. This article further discusses how the logo serves not only as a visual identity but also as a cultural and professional representation of the region’s collective progress.

### Advancing the Balkan Clinical Laboratory Federation (BCLF)

The conception of the Balkan Clinical Laboratory Federation emerged during the farewell reception of the IFCC Clinical Laboratory Management Course in May 1992, held in Sofia. The foundational ideas were initially proposed by Professor Danev (Sofia), Professor Terzioglu (Smyrna), Dr. Jullien (Athens), Dr. Buzo (Tirana), Professor Shipkov (Sofia), and Assistant Professor Tzontcheva (Sofia). These ideas were subsequently refined and further developed through the insightful contributions of the distinguished visiting IFCC lecturer, Professor P. Broughton. After approximately one year of extensive and dynamic correspondence among key individuals and institutions in Sofia, Birmingham, Athens, Istanbul, Smyrna, Skopje, and Bucharest, the inaugural meeting of the Balkan Clinical Laboratory Federation was organized in Sofia at the Centre of Hygiene. This event was orchestrated by Professor J. Todorov, Honorary President and Founding Member of Bulgarian Clinical Laboratory Medicine, marking a pivotal moment in establishing the Federation [5] [6]. The first meetings of the BCLF, which took place from 1993 to 1996 included three sessions held in Bulgaria, Sofia, Istanbul, and Struga [7].

The BCLF is a regional entity that unites all the countries of the Balkan Peninsula, specifically Albania, Bosnia and Herzegovina, Bulgaria, Greece, North Macedonia, Montenegro, Romania, Serbia, and Turkey (The countries included in the BCLF federation, listed in alphabetical order). These nations are affiliated with prominent international organizations, namely the European Federation of Clinical Chemistry and Laboratory Medicine (EFLM) and the International Federation of Clinical Chemistry and Laboratory Medicine (IFCC), further emphasizing their global commitment to advancing clinical laboratory science [5] [6]. Among the scientists who have contributed most significantly to the current level of the BCLF, we find notable figures such as Prof. Dr. Nada Majkić-Singh, Prof. Dr. Tomris Ozben, and Prof. Dr. Nazmi Özer from Turkey, Prof. Dr. George Benga from Romania, and Prof. Dr. Anyla Bulo Kasneci from Albania, Prof. Dr. Jozo ]orić Bosnia Herzegovina, These individuals, along with others from Bosnia and Herzegovina, Dobrin Svinarov Bulgaria, Orestes Tsolas and Eleni Bairaktari from Greece, Katerina Tosheska-Trajkovska North Macedonia, Tanja Antunovic from Montenegro, have each played a crucial role in representing their respective countries within the BCLF. Their dedication and efforts have been instrumental in the federation’s success and development. The development of the BCLF to this point has been made possible by the contributions of many individuals, including our esteemed professors and colleagues, whose names may have been unintentionally overlooked. We sincerely hope they will forgive us for this omission [5] [6].

BCLF federation brings together Balkan countries for scientific activities to foster collaboration,enhance research capabilities, and promote innovation in this geographic region. As a regional organization, the BCLF unites clinical biochemists and molecular biochemists across the Balkan region. Its primary goal is to enhance clinical laboratory practices in each member country. This improvement is driven by ongoing medical discoveries and the introduction of novel technologies in all the Balkan countries and Turkey. Additionally, the organization seeks to adapt to changes in the structure and proceed with laboratory support for clinical activities. To reach these objectives, BCLF will implement a comprehensive strategic plan. This plan will serve as a roadmap for collaboration and innovation among member states, ensuring that best practices are shared and adopted throughout the Balkan region and Turkey. Through these efforts, BCLF aims to elevate the standards of clinical laboratory services and ultimately improve patient care in the Balkans [5] [6].

The strategic objectives of the BCLF include enhancing member engagement, fortifying communication within the region and with international organizations such as the EFCC and IFCC, and establishing a dedicated BCLF journal or collaborating with established national publications. Furthermore, the BCLF aims to increase its visibility by organizing congresses, educational courses, and collaborative seminars with entities such as the IFCC, FESCC, WHO, and other relevant organizations. Additionally, the Federation is committed to advancing professional development, supporting research focusing on patient health, and fostering regional and international collaboration within the clinical laboratory community [5].

The establishment of this federation has led to the organization of meetings and congresses, ensuring that at least two representatives from each member country participate annually. The federation actively supports these initiatives, facilitating the dissemination of information regarding the events to researchers and scientists engaged in clinical biochemistry within each nation. This structured approach enhances collaboration and knowledge sharing among professionals in the field, fostering a robust network of expertise across the Balkan region [5] [6].

### Organizational structure and goals of the BCLF

The BCLF was established to address the growing need for professional unity and standardization in clinical laboratory practices in the Balkan geographic region. This federation became a problem-solving key player in clinical laboratory practices by promoting collaboration among the Balkan region’s clinical biochemistry scientists. This monograph will reveal the role of the BCLF, highlighting its impact on laboratory methods, and laboratory practices, broader the goals of the federation, and finally the impact on healthcare systems in the interregional cooperation within the Balkan countries. BCLF federation brings together clinical laboratories, medical professionals, and institutions from the Balkan and various other countries for instance the United States of America and Italy to foster knowledge exchange, method sharing support professional development, and improve the quality of laboratory services. Its founding was motivated by the shared historical, cultural, and healthcare challenges faced by the countries of the Balkan region [5] [6].

### Mission and goals of the BCLF

The BCLF is focused on several key goals that shape its mission. The BCLF is crucial in ensuring that laboratory results are comparable across different times, locations, and measurement procedures, essential for effective patient care, clinical research, and public health initiatives. This comparability is achieved through metrological traceability, a process that guarantees measurement procedures assess the same quantity and that their calibration is linked to a common reference system, which includes recognized reference methods and materials. While standardization ensures traceability to the International System of Units (SI), harmonization, as promoted by the BCLF, focuses on aligning measurement procedures with a reference system that is widely accepted by international consensus. Through these efforts, the BCLF helps maintain consistency and accuracy in laboratory testing across the region [5] [6].

Standardization of clinical laboratory tests is one of the key goals of the BCLF to establish common standards and guidelines for laboratory procedures across the region, ensuring that diagnostic results are consistent and reliable no matter where they are performed. This is made possible through the clinical biochemistry data shared at BCLF conferences, where experts gather to exchange knowledge and discuss the best clinical laboratory test methods, and standardized laboratory methods across the Balkans.

The BCLF is dedicated to organizing educational events, conferences, and workshops that help laboratory professionals grow in their knowledge and skills. These efforts play a vital role in improving the overall quality of laboratory medicine across the Balkans, ensuring that professionals stay up to date with the latest advancements in the field.

Another important part of the BCLF’s work is advocating for better healthcare policies, especially laboratory services. The federation actively represents the needs of clinical laboratories both nationally and internationally, working to drive reforms and secure more funding for laboratory infrastructure. This advocacy ensures that laboratory services receive the attention and support they need to thrive.

A key aspect of the BCLF’s work is advocating for improved healthcare policies, especially laboratory services. The federation represents the interests of clinical laboratories at both national and international levels, advocating for essential reforms and increased funding to enhance laboratory infrastructure. This ensures that laboratory services are provided with the essential resources and support needed to effectively address the changing requirements of the healthcare system in the region.

The BCLF encourages the exchange of knowledge by organizing presentations from scientists who apply new methodologies at its conferences. This allows researchers from across the Balkan region to learn about the latest advancements and incorporate these innovative approaches into their work, ultimately improving the quality of clinical laboratory medicine [5] [6].

### The conceptualization and symbolism of the BCLF logo

**Figure 1:**
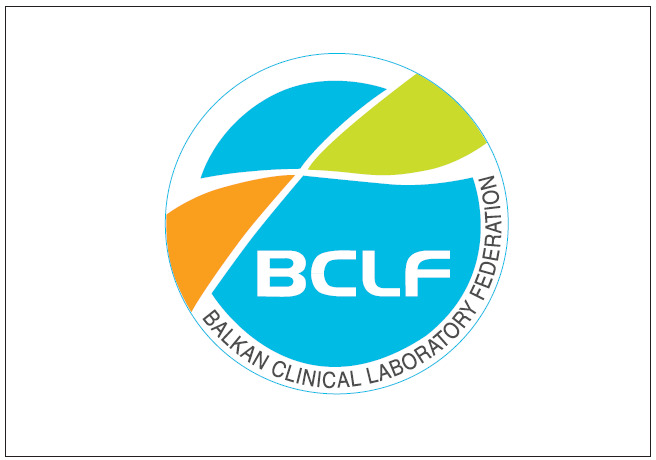
The logo of the Balkan Clinical Laboratory Federation (BCLF).

The logo of BCLF holds significant meaning, encapsulating the organization’s core values and mission in a visually compellingly. Each color and each shape carry meaning; this meaning is both symbolic and laden with significance. Upon closer examination of this logo, the blue represents the sea, while the orange portion symbolizes the Balkans. The green section represents Turkey, collectively illustrating the interconnectedness of the region. The selection of the logo took place during a BCLF congress. The choice of this logo for BCLF was not a straightforward process, as each participating country of the federation proposed its logo. My colleague Prof. Dr. Mustafa Serteser and me Prof. Dr. Nuriye Nuray Ulusu were informed that we would attend this meeting, where we are scheduled to present our research. At that time, we were at the beginning of our academic careers, and neither were professors. We were extremely excited about the opportunity to participate in such an event. Professor Dr. Nazmi Özer emphasized the importance of colors and shapes in effectively advocating for the logo during our preparation Armed with this knowledge, we vigorously defended Turkey’s proposed logo throughout the meeting. There were other suggestions and logos also in the meeting for instance. Figure 1

At the Executive Board members meeting, there were several alternative suggestions, though many were far removed from being suitable logos or logos. For instance, some proposals included a Balkan and Turkey region map, another example is the use of DNA symbols within the letters of BCLF. Another suggestion was a droplet of blood from a pipette. The DNA proposal was rejected because it did not form a fundamental aspect of clinical biochemistry. If I remember correctly, Prof. Dr. George Benga was the president of the association representing his country and he suggested the entire Balkan countries map of the countries as the logo of BCFL. The map was drawn in black on a white background. However, this map also was found unsuitable as it was too small, invisible, and overly complex when scaled down. Lastly, the blood droplet suggestion was dismissed due to its connotations of warfare and the resemblance of the pipette to a weapon. On the other hand, all the logos, except for the one suggested by Turkey, were drawn in black on a white background. Turkey’s proposed logo has faced criticism in contemporary discourse, particularly regarding its colorful design. Critics have pointed out the high cost of color photocopying and color prints. In response to this critique, we suggested that the price of color photocopying and color prints is likely to become cheaper over time. We have also mentioned that it is possible to use a black-and-white version if preferred. The black-andwhite photocopy of the map also looked visually appealing, and it was highly valuable because it symbolized the connection and unity of all the Balkan countries, including Turkey, which has territory in the Balkans [7].

The logo of the BCLF began at the BCLF congress in Tirana in 2010 and was accepted in Romania in 2011. This beautiful logo symbolizes a specific geographic area; the most important aim is creating novel knowledge, connection, and ongoing vitality. The Executive Board members’ efforts are to continue. On the other hand, the selection of the orange and the green colors may not be coincidental. Orange embodies qualities such as optimism, and enthusiasm while green represents new beginnings and growth. The sea symbolizes grandeur, expansiveness, infinity, depth, and abundance [8].

The BCLF logo shows the creation of novel clinical biochemistry research optimism, and enthusiasm while conveying a sense of infinity and depth.

In conclusion, integrating the community into innovation and research on clinical laboratory medicinal biochemistry will benefit Turkey and the Balkan countries contribute to the global scientific community, and foster a shared vision of progress and collaboration.

The BCLF association has brought together the representatives of the countries to facilitate scientific exchanges, where they guide young professionals in clinical biochemistry and even basic biochemistry. This collaboration has contributed to elevating clinical biochemistry in the Balkan region to a level that is now a source of pride.

## Conclusion

The BCLF has played a crucial role in shaping the landscape of clinical laboratory medicine acrossthe Balkan region. Through its commitment to collaboration, education, and research, the BCLF has significantly enhanced the quality of laboratory practices and fostered innovation in the field. The creation of the BCLF logo, with its meaningful symbolism, reflects the federation’s mission of unity, progress, and shared commitment to advancing healthcare in the region. By promoting standardization, supporting research, and advocating for improved healthcare policies, the BCLF has made a lasting impact on the clinical laboratory profession. The federation’s efforts to connect professionals from diverse backgrounds, share knowledge, and drive change continue to strengthen the clinical laboratory community in the Balkans. As the BCLF moves forward, its role inadvancing clinical biochemistry and fostering regional and international collaboration will remain essential to healthcare and scientific progress in the Balkans and beyond.

## Dodatak

### Ethical approval

No ethical approval is required for this monograph.

### Authors contributions

N. Nuray Ulusu and Nada Majkić-Singh were responsible for conceptualizing and writing the original manuscript and revisions.

Equally contributed N. Nuray Ulusu and Nada Majkić-Singh.

### Funding

No funding.

### Competing interests

N. Nuray Ulusu and Nada Majkić-Singh declare no conflict of interest.

### Conflict of interest statement

All the authors declare that they have no conflict of interest in this work.
